# Effect of guselkumab on serum biomarkers in patients with active psoriatic arthritis and inadequate response to tumor necrosis factor inhibitors: results from the COSMOS phase 3b study

**DOI:** 10.1186/s13075-023-03125-4

**Published:** 2023-08-16

**Authors:** Georg Schett, Warner Chen, Sheng Gao, Soumya D. Chakravarty, May Shawi, Frederic Lavie, Miriam Zimmermann, Mohamed Sharaf, Laura C. Coates, Stefan Siebert

**Affiliations:** 1 Department of Medicine 3 – Rheumatology and Immunology, Universitätsklinikum Erlangen, Erlangen, Germany; 2 Immunology, Janssen Research & Development, LLC, Spring House, PA USA; 3 Immunology, Janssen Scientific Affairs, LLC, Horsham, PA USA; 4 Drexel University College of Medicine, Philadelphia, PA USA; 5 Immunology, Global Medical Affairs, Janssen Pharmaceutical Companies of Johnson & Johnson, Horsham, PA USA; 6 Immunology, Global Medical Affairs, Janssen Pharmaceutical Companies of Johnson & Johnson, Issy Les Moulineaux, France; 7 Immunology, Janssen Medical Affairs, LLC, Zug, Switzerland; 8 Johnson & Johnson MEA, Dubai, United Arab Emirates; 9 Nuffield Department of Orthopaedics, Rheumatology and Musculoskeletal Sciences, University of Oxford, Oxford, UK; 10 School of Infection and Immunity, University of Glasgow, Glasgow, UK

**Keywords:** Serum biomarkers, IL-23/IL-17 pathway, Guselkumab, Psoriatic arthritis

## Abstract

**Background:**

Guselkumab is a selective interleukin (IL)-23 inhibitor targeting the IL-23p19 subunit. In the phase 3b COSMOS trial, guselkumab demonstrated efficacy in treating participants with active psoriatic arthritis (PsA) and inadequate response (IR; lack of efficacy or intolerance) to tumor necrosis factor inhibitors (TNFi).

**Methods:**

Adults with active PsA (≥ 3 swollen joints,  ≥ 3 tender joints) and IR to one or two TNFi (TNFi-IR) were randomized 2:1 to guselkumab at Weeks 0, 4, then every 8 weeks (Q8W) or placebo➔guselkumab Q8W at Week 24 with possible early escape at Week 16. Levels of serum cytokines, including interferon γ (IFNγ), IL-10, and tumor necrosis factor α (TNFα); T helper 17 (Th17) effector cytokines IL-17A, IL-17F, and IL-22; and acute phase proteins C-reactive protein (CRP), IL-6, and serum amyloid A (SAA), were assessed and compared with demographically matched healthy controls; guselkumab pharmacodynamics through Week 24 were also assessed. Associations between baseline biomarker levels and 1) baseline disease activity (28-joint disease activity score using CRP [DAS28-CRP], psoriasis area and severity index [PASI], and % body surface area [BSA] affected by psoriasis) and 2) clinical response (including  ≥ 20% improvement in American College of Rheumatology criteria [ACR20] response) at Week 24 were assessed.

**Results:**

Baseline serum levels of IL-6, IL-10, IL-17A, IL-17F, IL-22, TNFα, and IFNγ were significantly higher in COSMOS TNFi-IR participants than in healthy controls. Baseline IL-6, CRP, and SAA levels were associated with baseline DAS28-CRP. IL-17A and IL-17F levels were associated with baseline PASI score and psoriasis BSA. Baseline swollen or tender joint counts did not associate with baseline biomarker levels. At Week 24, significant decreases from baseline in CRP, SAA, IL-17A, IL-17F, and IL-22 levels were seen in guselkumab-, but not placebo-, treated participants. IL-17F and IL-22 levels at Week 24 in guselkumab-treated participants did not significantly differ from those of healthy controls. Guselkumab-treated participants achieving ACR20 response at Week 24 exhibited higher baseline IL-22 and IFNγ levels versus nonresponders.

**Conclusions:**

Results from COSMOS participants with active, TNFi-IR PsA suggest guselkumab reduces levels of effector cytokines associated with the IL-23/IL-17 pathway, including those associated with baseline arthritis and skin disease activity.

**Trial registration:**

ClinicalTrials.gov: NCT03796858.

**Supplementary Information:**

The online version contains supplementary material available at 10.1186/s13075-023-03125-4.

## Background

Psoriatic arthritis (PsA) is a heterogenous and chronic systemic inflammatory disease that can manifest in multiple domains [1, 2]. Tumor necrosis factor inhibitors (TNFi) are often prescribed for patients with PsA who do not have an adequate response to conventional therapies [3, 4]. However, approximately 40% of patients do not achieve  ≥ 20% improvement in the American College of Rheumatology criteria (ACR20) response within 6 months of treatment of their first TNFi [5]. Furthermore, treatment effectiveness and persistence have been shown to decline with successive TNFi [6–8]. Treatments targeting alternative pathways have been evaluated and shown to be efficacious in TNFi-experienced patients [9–12], suggesting that this population may benefit from therapies with different mechanisms of action. Treatment recommendations from the Group for Research and Assessment of Psoriasis and Psoriatic Arthritis have highlighted interleukin (IL)-23 inhibitors (IL-23i), along with TNFi, IL-17i, and IL-12/IL-23i, as appropriate for use in both biologic-naïve and biologic-experienced patients with peripheral joint symptoms [4].

Guselkumab is a high-affinity, fully human, monoclonal antibody that targets the IL-23p19 subunit [13, 14]. In the phase 3 DISCOVER-1 (ClinicalTrials.gov Identifier: NCT03162796) and DISCOVER-2 (ClinicalTrials.gov Identifier: NCT03158285) trials, guselkumab demonstrated significant efficacy in participants with active PsA [13, 14]. Both studies enrolled patients with PsA who were biologic-naïve, and DISCOVER-1 also included patients (31% of randomized participants) who had previously received one or two TNFi. Moreover, in the phase 3b COSMOS trial (ClinicalTrials.gov Identifier: NCT03796858), guselkumab was shown to be efficacious in reducing disease signs and symptoms in participants with an inadequate response (IR), characterized as lack of efficacy or intolerance, to TNFi (TNFi-IR) [15].

Previous analyses have shown that C-reactive protein (CRP) and type 17 effector cytokine serum levels are elevated in patients with PsA and can be reduced by inhibiting the IL-23p19 subunit or the IL-12/23p40 subunit [16, 17]. The objectives of this analysis were to evaluate baseline serum levels of proinflammatory biomarkers in participants with TNFi-IR PsA in the COSMOS trial in comparison with healthy controls and the relationship of these biomarker levels with baseline disease activity. Analyses also aimed to evaluate changes in biomarker levels with guselkumab versus placebo and to assess the association of clinical response with biomarker levels over time.

## Methods

### Study design, participants, and endpoints

The study design and participant eligibility criteria for COSMOS have been previously described [15]. In brief, COSMOS was a phase 3b, randomized, double-blind trial in adults with PsA (per ClASsification criteria for Psoriatic ARthritis [CASPAR]) who had active disease (≥ 3 swollen and  ≥ 3 tender joints), active (≥ 1 psoriatic plaque of  ≥ 2 cm) or documented history of plaque psoriasis, and a history of IR (lack of efficacy or intolerance) to one or two TNFi. Eligible participants were randomized (2:1) to receive subcutaneous injections of either guselkumab 100 mg (Week 0, Week 4, and then every 8 weeks [Q8W]) or matching placebo with crossover to guselkumab 100 mg at Week 24, Week 28, and then Q8W. The final dose of study agent was administered at Week 44. Participants with  < 5% improvement from baseline in tender joint count (TJC) and swollen joint count (SJC) at Week 16 qualified for early escape, with guselkumab-treated participants continuing randomized treatment and placebo-treated participants crossing over to receive guselkumab 100 mg at Week 16, Week 20, and then Q8W. COSMOS was conducted in accordance with the Declaration of Helsinki and Good Clinical Practice guidelines, and all participants provided written informed consent. The trial protocol was approved by the governing ethical body at each site.

### Clinical assessments

Among participants in the biomarker cohort, baseline disease activity was evaluated using SJC, TJC, 28-joint Disease Activity Score using CRP (DAS28-CRP) [18], Psoriatic Arthritis Disease Activity Score (PASDAS) [19], Disease Activity index for Psoriatic Arthritis (DAPSA) [20], Psoriasis Area and Severity Index (PASI) [21], and body surface area (BSA) affected by psoriasis. ACR20 response was used to assess efficacy in the joints at Week 24. Skin responses were assessed among participants with  ≥ 3% BSA and Investigator’s Global Assessment (IGA) score ≥ 2 [22] at baseline and included IGA 0/1 response (defined as IGA score of 0 or 1 and  ≥ 2-grade improvement from baseline) at Week 24. To assess consistency of response in the biomarker cohort versus the overall COSMOS population, additional efficacy outcomes at Week 24 determined in the biomarker cohort were ≥ 50% improvement by ACR criteria (ACR50) response,  ≥ 75% improvement in PASI (PASI75), change from baseline in Health Assessment Questionnaire–Disability Index (HAQ-DI) score [23], and change from baseline in the 36-item Short-Form Health Survey physical component summary (SF-36 PCS) score [24].

### Biomarker sample collection

In COSMOS, blood samples for biomarker analyses were collected from all participants at Weeks 0, 4, 16, 24, and 48 into standard serum separation tubes. After 30 min, serum was separated via centrifugation at room temperature (15–20 min at 1500 × *g*) and then subsequently aliquoted and stored at −20 °C. Biomarker data were retrospectively generated for a subgroup of participants (biomarker cohort). The total sample size was chosen to align with prior biomarker analyses from the DISCOVER-1 and -2 trials. The primary criteria for selection into the biomarker cohort were based on the availability of participant samples, treatment group, and clinical response defined by multiple endpoints. To avoid inadvertently skewing selection of samples for any known demographic or clinical characteristic, a representative subgroup of samples was selected to reflect the overall trial population based on availability of biomarker samples at all three time points of interest, treatment group, clinical response (ACR20), prior TNFi treatment, and demographics.

Serum samples from 24 healthy control volunteers (defined as those with no signs of active inflammation, PsA, or psoriasis based on physical assessment, medical history, and current medication) were also assessed for biomarkers. These samples were procured independently from a third party (Bioreclamation, Westbury, NY; Biological Specialty Corp., Colmar, PA).

### Biomarker analyses

Serum samples for biomarker analyses were analyzed using qualified antibody-based assays. Serum concentrations of the T helper 17 (Th17) effector cytokine IL-17A were analyzed using Simoa™ single molecule array technology (Quanterix Corp., Billerica, MA), and those of IL-17F and IL-22 were analyzed using the Single Molecule Counting SMCxPRO Immunoassay Platform (Millipore, Burlington, MA). Acute phase proteins and markers of inflammation, CRP, serum amyloid A (SAA), IL-6, IL-10, interferon γ (IFNγ), tumor necrosis factor α (TNFα), soluble intercellular adhesion molecule-1 (sICAM), and vascular cell adhesion molecule (sVCAM), were analyzed using the Meso Scale Discovery Platform (Meso Scale Diagnostics, Rockville, MD).

### Statistical analysis

This analysis included participants with available baseline values and follow-up biomarker and clinical data over time. All analyses were post hoc; thus, reported *p* values are nominal.

#### Analysis of treatment effect on clinical efficacy

Differences in efficacy outcomes evaluated in the COSMOS biomarker cohort with guselkumab versus placebo treatment were assessed using a Chi-square test (categorical outcomes) or ANOVA (continuous measures).

#### Analysis of baseline serum biomarker levels and correlation with disease activity

Differences in baseline serum cytokine levels between participants with PsA and healthy controls were assessed using log2-transformed data with a general linear model. Serum protein expression levels were log2-transformed to normalize the data distribution. Differences of  ≥ 1.4-fold with *p* < 0.05 were considered significant.

Correlations between baseline serum biomarker levels and baseline disease activity (i.e., DAPSA scores, PASDAS, and PASI scores) were assessed using Spearman linear regression, with a Spearman correlation (*rho*)  > 0.25 and *p* < 0.05 considered significant.

#### Analysis of treatment effect on biomarker levels

For the evaluation of treatment effects (pharmacodynamic responses), changes in biomarker levels were compared between the active treatment and placebo groups. A contrast dataset for within-participant changes in biomarkers was generated from log2-transformed data, with the difference between the time point and baseline calculated for each participant and time point (Week 4, 16, 24, and 48). Differences were considered significant if they were  ≥ 1.4-fold with a *p* < 0.05.

#### Analysis of association between biomarker levels and clinical response

Differences in baseline biomarker levels by clinical response at Week 24 (i.e., response versus nonresponse for ACR20/50, IGA 0/1, and PASI75) were evaluated using general linear model analyses and log2-transformed biomarker levels. Clinical response categorical variable was set as the primary fixed factor. In determining response, participants with missing efficacy data were considered nonresponders (nonresponder imputation). To evaluate the significance of changes in biomarkers from baseline among clinical responders and nonresponders, levels were compared between the specified time point and baseline separately for each clinical response group using general linear model analyses. Visit was set as the primary fixed variable.

## Results

### COSMOS participants and COSMOS biomarker cohort

In COSMOS, 189 participants with active TNFi-IR PsA were randomized to receive guselkumab Q8W, and 96 participants were randomized to receive placebo followed by guselkumab. Of these, 100 and 50 participants, respectively, were included in the biomarker cohort. In the COSMOS biomarker cohort, 21/100 (21%) participants in the guselkumab Q8W arm and 24/50 (48%) participants in the placebo arm qualified for early escape at Week 16.

Baseline demographics and disease characteristics were generally similar between the overall COSMOS population and the biomarker cohort (Supplementary Table 1). Participant characteristics were also generally consistent between treatment groups in the biomarker cohort, although several small numerical differences in the proportion of females, measures of skin and joint disease, and duration of PsA may suggest that participants in the guselkumab group represent a somewhat more difficult-to-treat subgroup than those in the placebo group (Supplementary Table 1).

### Clinical efficacy in the COSMOS biomarker cohort

Among participants in the biomarker cohort, 44% in the guselkumab group versus 20% in the placebo group achieved an ACR20 response at Week 24 (*p* < 0.01), consistent with the significant treatment effect observed in the overall study population [15]. Also consistent with the overall COSMOS study, guselkumab-treated participants in the biomarker cohort demonstrated significant treatment effects versus placebo at Week 24 across several secondary endpoints and disease domains (changes from baseline in HAQ-DI and SF-36 PCS scores; IGA 0/1 and PASI75 response rates) compared with placebo (Supplementary Table 2).

### Biomarker analyses

#### Baseline serum levels and correlation with baseline disease activity

**Fig. 1:**
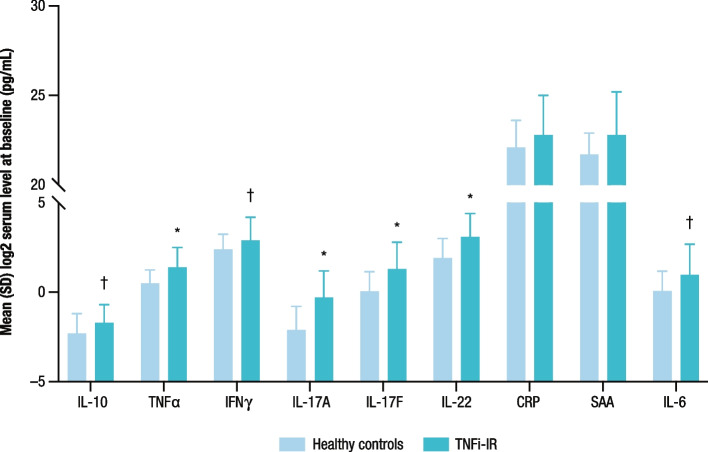
Baseline levels of serum cytokines (IL-10, TNFα, and IFNγ), Th17 effector cytokines (IL-17A, IL-17F, and IL-22) and acute phase proteins (CRP, SAA, and IL-6) in participants with TNFi-IR PsA from COSMOS and in healthy controls. ^*^p < 0.001 TNFi-IR PsA versus HCs; ^†^*p* < 0.05 TNFi-IR PsA versus HCs. CRP, C-reactive protein; HC, healthy control; IFN, interferon; IL, interleukin; PsA, psoriatic arthritis; SAA, serum amyloid A; SD, standard deviation; Th17, T helper 17; TNF, tumor necrosis factor; TNFi-IR, tumor necrosis factor inhibitor inadequate response

Baseline serum concentrations of IL-6, IL-10, IL-17A, IL-17F, IL-22, TNFα, and IFNγ were significantly higher in the COSMOS biomarker cohort compared with healthy controls (Fig. 1). In addition, concentrations of several biomarkers were significantly correlated with at least one measure of clinical disease activity at baseline. Baseline CRP, SAA, and IL-6 levels were positively associated with baseline joint disease severity as measured by the DAS28-CRP (*p* ≤ 0.0001 for each biomarker). Baseline CRP levels positively correlated with baseline PASDAS (*p* = 0.0014), and IL-6 and SAA levels trended toward a correlation with PASDAS (Table 1). Baseline biomarker levels did not correlate with baseline DAPSA (including CRP) score, SJC, or TJC. Statistically significant correlations were also observed between baseline IL-17A, IL-17F, and IL-22 levels and baseline PASI score as well as between IL-17A and IL-17F levels and BSA affected by psoriasis. IL-17A levels showed a trend towards a correlation with PASDAS. No statistically significant correlations were observed between baseline levels of other biomarkers evaluated and measures of baseline disease activity assessed (Table 1).

**Table 1:** Correlation of baseline cytokine biomarker levels with baseline clinical activity measures in participants with TNFi-IR PsA in the COSMOS biomarker cohort

	**Clinical activity in the COSMOS biomarker cohort**								
**Biomarker**		**PsA duration**	**PsO BSA**	**PASI** **(0–72)**	**SJC** **(0** *–* **66)**	**TJC** **(0** *–* **68)**	**DAS28-CRP**	**PASDAS**	**DAPSA**
**CRP**	*Rho*	*0.12*	*0.09*	*0.08*	*-0.04*	*0.03*	***0.39***	***0.26***	*0.12*
	*p* value	0.128	0.274	0.325	0.640	0.743	** < 0.0001**	**0.0014**	0.1330
**SAA**	*Rho*	*0.09*	*0.08*	*0.04*	*0.03*	*0.04*	***0.32***	*0.22*	*0.15*
	*p* value	0.266	0.349	0.620	0.721	0.634	**0.0001**	0.0075	0.0683
**IL-6**	*Rho*	*0.24*	*-0.01*	*-0.02*	*0.06*	*0.12*	***0.32***	*0.24*	*0.18*
	*p* value	0.004	0.944	0.834	0.499	0.149	**0.0001**	0.0029	0.0277
**IL-17A**	*Rho*	*0.07*	***0.48***	***0.49***	*0.16*	*0.06*	*0.18*	*0.22*	*0.13*
	*p* value	0.399	** < 0.0001**	** < 0.0001**	0.045	0.446	0.029	0.0076	0.1202
**IL-17F**	*Rho*	*0.21*	***0.44***	***0.41***	*0.14*	*0.0*	*0.10*	*0.14*	*0.10*
	*p* value	0.009	** < 0.0001**	** < 0.0001**	0.086	0.265	0.219	0.0850	0.2189
**IL-22**	*Rho*	*0.12*	*0.21*	***0.25***	*–0.06*	*0.00*	*0.04*	*–0.19*	*0.24*
	*p* value	0.131	0.0091	**0.0024**	0.4862	0.961	0.6532	0.0559	0.0147
**IFNγ**	*Rho*	*0.1068*	*0.0214*	*0.0076*	*–0.0112*	*0.0034*	*0.0435*	*0.07*	*0.02*
	*p* value	0.1935	0.7949	0.9263	0.8916	0.9674	0.5968	0.3785	0.8391
**IL-10**	*Rho*	*0.0776*	*0.0627*	*0.0718*	*0.0132*	*0.0141*	*–0.0356*	*0.04*	*0.01*
	*p* value	0.3453	0.446	0.3824	0.8729	0.864	0.6651	0.6139	0.8901
**IL-8**	*Rho*	*0.2004*	*0.1104*	*0.0578*	*–0.0268*	*0.0486*	*0.0846*	*0.16*	*0.05*
	*p* value	0.0139	0.1787	0.4823	0.7448	0.5548	0.3031	0.0496	0.536
**TNFα**	*Rho*	*0.213*	*0.0032*	*0.0371*	*–0.0445*	*–0.014*	*0.0416*	*0.07*	*0.01*
	*p* value	0.0089	0.9688	0.6523	0.5888	0.8646	0.6125	0.4211	0.928
**sICAM-1**	*Rho*	*0.0678*	*0.0067*	*0.0599*	*-0.0512*	*-0.0089*	*0.1282*	*0.08*	*0.02*
	*p* value	0.4097	0.9349	0.4664	0.5342	0.9137	0.1179	0.354	0.7711
**sVCAM-1**	*Rho*	*0.1155*	*–0.0325*	*–0.0414*	*0.0992*	*0.1499*	*0.101*	*0.13*	*0.16*
	*p* value	0.1594	0.6928	0.6148	0.2271	0.067	0.2186	0.1227	0.0568

Statistics based on Spearman linear regression

Bolded text highlights significant correlation between biomarker and clinical activity (*Rho* > 0.25; *p* < 0.05)

BSA, body surface area; CRP, C-reactive protein; DAPSA, Disease Activity index for Psoriatic Arthritis; DAS28-CRP, 28- Disease Activity Score with C-reactive protein; IL, interleukin; PASDAS, Psoriatic Arthritis Disease Activity Score; PASI, Psoriasis Area and Severity Index; PsA, psoriatic arthritis; PsO, psoriasis; SAA, serum amyloid A; sICAM, soluble intercellular adhesion molecule-1; SJC, swollen joint count; sVCAM, soluble vascular cell adhesion molecule; TJC, tender joint count; TNF, tumor necrosis factor; TNFi-IR, tumor necrosis factor inhibitor-inadequate response

#### Effect of treatment on biomarker levels

**Fig. 2:**
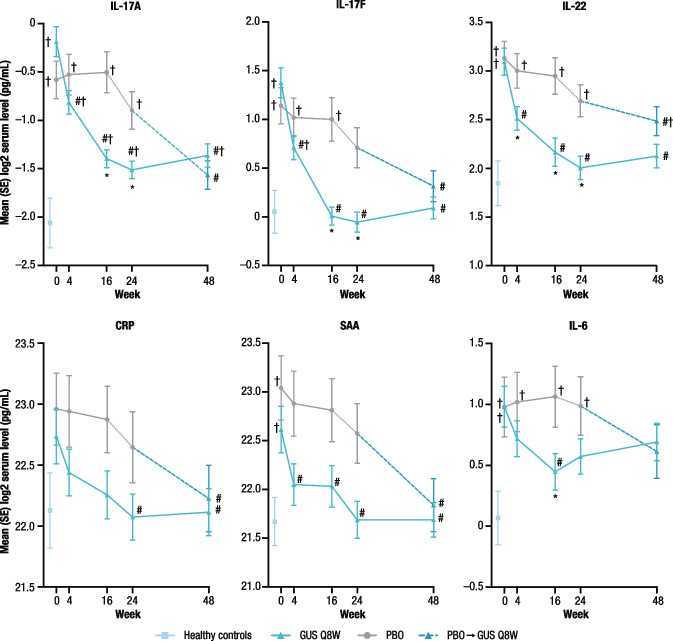
Serum levels of IL-17A, IL-17F, IL-22, CRP, SAA, and IL-6 in participants with TNFi-IR PsA from COSMOS compared with healthy controls over time. Participants randomized to placebo crossed over to guselkumab at Week 16 (early escape; dotted line; *n* = 24) or at Week 24 (per protocol; dashed line; *n* = 26). Statistics based on general linear model. Error bars represent 1 standard error. ^*^Indicates statistical significance versus placebo by *p* < 0.05 and |fold difference | ≥ 1.4. ^#^Indicates statistical significance versus baseline by *p* < 0.05 and |fold difference | ≥ 1.4. ^†^Indicates statistical significance versus healthy controls by *p* < 0.05 and |fold difference | ≥ 1.4. CRP, C-reactive protein; GUS, guselkumab; IFN, interferon; IL, interleukin; PBO, placebo; PsA, psoriatic arthritis; Q8W, every 8 weeks; SAA, serum amyloid A; TNFi-IR, tumor necrosis factor inhibitor-inadequate response

In participants randomized to guselkumab, reductions from baseline in levels of the Th17 effector cytokines IL-17A, IL-17F, and IL-22 and the acute phase proteins IL-6, CRP, and SAA were observed, while changes from baseline were not apparent in those who received placebo (Fig. 2). For IL-17A, IL-17F, IL-22, and SAA, the reductions from baseline in the guselkumab group were statistically significant by Week 4, continued through Week 16, and were sustained through Weeks 24 and 48. Serum levels of IL-17F (from Week 16), IL-22 (from Week 4), and IL-6 (at Week 16) were normalized in the guselkumab group, but not the placebo group, when compared with levels observed in healthy controls. Additionally, at Weeks 24 and 48, levels of CRP and SAA in the guselkumab group approximated those observed in healthy controls. For participants who crossed over from placebo to guselkumab at Weeks 16 or 24, reductions in serum biomarkers at Week 48 were similar to those observed in participants receiving guselkumab Q8W from baseline (Fig. 2).

#### Biomarker levels and clinical response

**Fig. 3:**
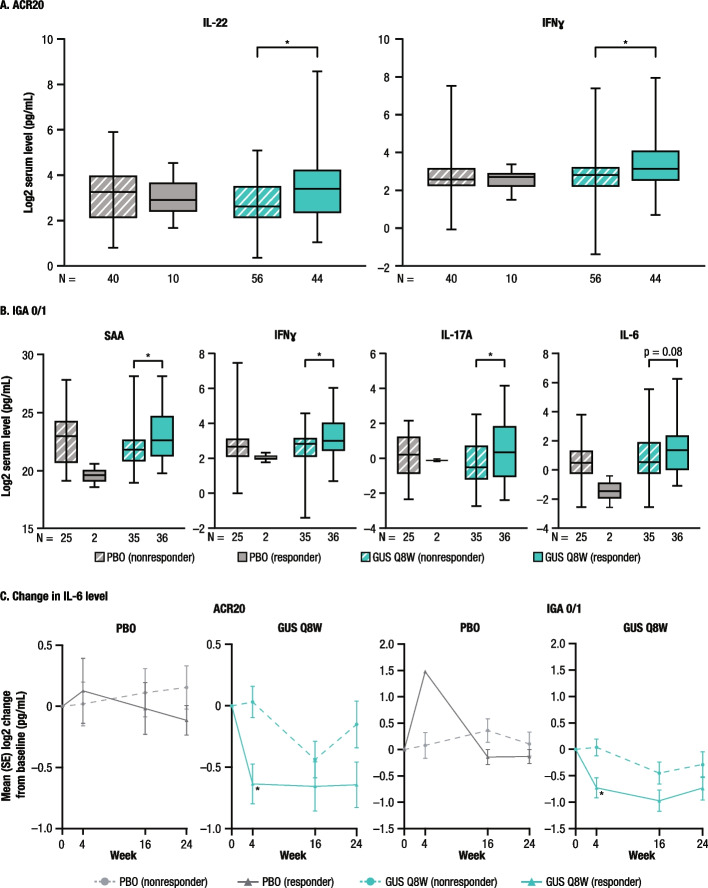
Baseline serum cytokine levels and clinical response at Week 24 in participants with TNFi-IR PsA from COSMOS by ACR20 response or IGA 0/1 response and change from baseline in serum IL-6 level by ACR20 or IGA 0/1 response over time. (**A**) Baseline serum levels of IL-22 and IFNγ levels by ACR20 response at Week 24; (**B**) Baseline serum levels of SAA, IFNγ, IL-17A, and IL-6 by IGA 0/1 response at Week 24; (**C**) Change from baseline in IL-6 levels by ACR20 and IGA 0/1 responses through Week 24. ^*^p < 0.05 between responders and nonresponders. ACR20,  ≥ 20% improvement in American College of Rheumatology response criteria; GUS, guselkumab; IFN, interferon; IGA 0/1 response, Investigator’s Global Assessment score of 0 or 1 with at least a 2-point improvement from baseline; IL, interleukin; PBO, placebo; PsA, psoriatic arthritis; Q8W, every 8 weeks; SAA, serum amyloid A; TNFi-IR, tumor necrosis factor inhibitor-inadequate response

Participants in the guselkumab group, but not the placebo group, who achieved an ACR20 response at Week 24 exhibited higher baseline IL-22 and IFNγ levels compared with ACR20 nonresponders (Fig. 3A). Additionally, among participants in the guselkumab group, baseline serum levels of SAA, IFNγ, and IL-17A were significantly higher in participants achieving versus not achieving an IGA 0/1 response at Week 24 (Fig. 3B). Baseline levels of SAA, IFNγ, and IL-17A were similar among participants in the placebo group, regardless of IGA 0/1 response or nonresponse at Week 24. No other significant associations between baseline biomarker levels and clinical response at Week 24 were observed in the guselkumab group (data not shown).

Participants in the guselkumab group who achieved an ACR20 response at Week 24 exhibited a significantly greater reduction in IL-6 level from baseline at Week 4 compared with ACR20 nonresponders (Fig. 3C). Similarly, IGA 0/1 responders in the guselkumab group also achieved a significantly greater early (Week 4) reduction in IL-6 compared with nonresponders. In contrast, no significant changes in IL-6 levels were observed through Week 24 in placebo-treated participants, regardless of whether they were ACR20 or IGA 0/1 responders or nonresponders.

## Discussion

These results provide further support for the important role of the IL-23/IL-17 pathway in PsA pathogenesis and expand our knowledge of guselkumab pharmacodynamic effects in patients with TNFi-IR PsA. Baseline levels of IL-6, IL-10, IL-17A, IL-17F, IL-22, TNFα, and IFNγ were higher in participants with TNFi-IR PsA compared with healthy controls. These findings were generally consistent with results from an exploratory biomarker analysis [16] in patients with active PsA from the DISCOVER-1 and -2 trials [13, 14]. Both the prior and current analyses also found a significant correlation between baseline biomarker levels and baseline psoriasis disease activity (as assessed by both BSA and PASI) and baseline joint disease severity (as assessed by the DAS28-CRP). Taken together, observations to date indicate consistent pharmacodynamic effects of guselkumab in both patients with PsA who are biologic-naïve (85% of participants across the pooled DISCOVER trials) [16] and those who are TNFi-IR (COSMOS). It is important to note, however, that the DAS28-CRP composite measure was developed to assess disease activity in rheumatoid arthritis and its utility in PsA is limited [25]. Moreover, positive correlation with baseline DAS28-CRP and CRP levels was anticipated, as CRP level is included in the composite score calculation.

This study further demonstrates serum biomarker levels are likely associated with disease activity assessed using PsA-specific composite indices. For example, CRP levels positively correlated with PASDAS, which includes CRP level as a component. Further, IL-6, SAA, and IL-17A levels showed a trend towards correlation with PASDAS, suggesting their important roles in PsA disease activity. Also of note, a correlation between CRP and DAPSA was not observed despite DAPSA including CRP as a measure; the reason for this is unclear. In both the prior and current studies, the association between the serum biomarkers analyzed and SJC or TJC was limited and may indicate that tissue-specific variations in cytokine levels and/or other systemic factors also play a role in this complex disorder. Similar results were seen in analyses from the two phase 3 PSUMMIT studies of ustekinumab, which reported that serum levels of IL-23, IL-17A, and IL-17F correlated with baseline skin disease in participants with PsA, but did not find a clear association of these cytokines with joint disease [17].

Similar to the DISCOVER-1 and -2 biomarker results in participants who were mostly biologic-naïve [16], treatment with guselkumab Q8W, but not placebo, was associated with reductions from baseline in IL-17A, IL-17F, IL-22, and SAA as early as Week 4, IL-6 at Week 16 (Week 24 in DISCOVER-1 and -2), and CRP at Week 24. The present study extends these findings by demonstrating that the pharmacodynamic effect of guselkumab is sustained through Week 48 in the COSMOS TNFi-IR population, with levels of IL-17F, IL-22, CRP, and SAA approximating the levels seen in healthy controls at Week 48. In both biomarker studies, participants achieving clinical responses had higher mean baseline levels of some cytokines compared with nonresponders. These findings suggest that guselkumab is efficacious in patients with PsA who show a molecular signature of inflammation at baseline. These data also suggest that higher baseline levels of IL-22, SAA, IL-17A, and IFNγ may be predictive of clinical response to guselkumab treatment in the TNFi-IR PsA population. Furthermore, among participants in the guselkumab group who achieved ACR20 and IGA 0/1 responses at Week 24, significant reductions from baseline in IL-6 were observed as early as Week 4 versus nonresponders, suggesting that early changes in IL-6 expression in response to guselkumab may contribute to clinical responses at later time points. Despite the apparent normalization of Th17 effector cytokine levels with guselkumab treatment, other inflammatory cytokines beyond those in the IL-23/IL-17 pathway that play a role in PsA pathogenesis may contribute to residual disease symptoms.

Similar exploratory analyses have been performed in clinical studies with other classes of PsA treatments, including TNFi, IL-17i, and Janus kinase inhibitors [26–29]. Consistent with the findings here, elevated levels of serum inflammatory markers, such as CRP, SAA, and IL-6, have been associated with more active disease and poor prognosis [30–33]. Predictive serum biomarkers for treatment responses have also been reported [34–37]. For example, in PsA patients receiving the TNFi golimumab for active disease, higher baseline CRP levels were predictive of achievement of modified-minimal disease activity (mMDA) at 3 months, and were significantly associated with a higher probability of mMDA response at 6 months [38]. In PsA patients treated with IL-17i, baseline serum IL-22 levels were lower in those who achieved DAPSA remission compared with those who did not [39]. The current limited data on predictors of response across biomarker studies make it difficult to incorporate precision medicine in  the management of PsA at this stage. Thus, further studies are needed to better understand the involvement of inflammatory cytokines or acute phase reactants in PsA pathogenesis and the clinical utility of predictive biomarkers in guiding treatment choices.

Results of analyses reported herein are limited by the potential incongruence between serum and tissue (e.g., joint and skin) levels of cytokines and acute phase proteins. While analysis of serum levels allows for collection of serial samples in the clinic, evaluation of tissue would further our knowledge of disease pathogenesis and could potentially help further elucidate the mechanism(s) of action of guselkumab in joints, as preclinical evidence suggests that IL-17 is a key mediator of PsA joint pathogenesis [40]. The lack of an association between baseline IL-17 levels and ACR20 response raises the possibility that guselkumab has other pharmacodynamic effects that contribute to its efficacy in the treatment of PsA.

We have now consistently observed correlations between serum biomarker levels and PsA disease activity in the DISCOVER-1 and -2 and COSMOS trials that reinforce the important role of serum inflammatory factors and cytokines in PsA pathogenesis. Additional research is needed to confirm these findings in a TNFi-IR population and more fully contrast with bio-naïve PsA patients. Further research will also be needed to strengthen the potential of using serum inflammatory biomarker levels to guide therapeutic selection for patients with PsA.

## Conclusions

Overall, these data suggest that guselkumab reduces the levels of key effector inflammatory cytokines, including those associated with the IL-23/IL-17 pathway, at early time points in participants with TNFi-IR PsA. Reductions were generally sustained through Week 48, with levels of IL-17F, IL-22, CRP, and SAA approximating those observed in healthy controls. Higher baseline levels of certain cytokines and acute phase reactants were observed in ACR20 (IL-22 and IFNγ) and IGA 0/1 (SAA, IFNγ, and IL-17A) responders than in nonresponders at Week 24. The clinical utility and treatment implications of these findings require further investigation.

### Supplementary Information

**Additional file 1:** **Supplementary Table 1.** Baseline demographics and disease characteristics. **Supplementary Table 2. **Primary and secondary efficacy endpoint results in the COSMOS biomarker population.

## Data Availability

The data sharing policy of Janssen Pharmaceutical Companies of Johnson & Johnson is available at https://www.janssen.com/clinicaltrials/transparency. As noted on this site, requests for access to the study data can be submitted through Yale Open Data Access (YODA) Project site at http://yoda.yale.edu.

## Abbreviations

ACR20: ≥ 20% Improvement in American College of Rheumatology response criteria
ACR50: ≥ 50% Improvement in American College of Rheumatology response criteria
BSA: Body surface area
CASPAR: ClASsification criteria for Psoriatic ARthritis
CRP: C-reactive protein
DAS28-CRP: 28-joint Disease Activity Score with CRP
DAPSA: Disease Activity index for Psoriatic Arthritis
GUS: Guselkumab
HAQ-DI: Health Assessment Questionnaire–Disability Index
HC: Healthy control
IGA: Investigator’s Global Assessment
IGA 0/1 response: Investigator’s Global Assessment score of 0 or 1 with at least a 2-point improvement from baseline
IFN: Interferon
IL: Interleukin
IL-12/IL-23i: IL-12/IL-23 inhibitor
IL-17i: IL-17 inhibitor
IL-23i: IL-23 inhibitor
IR: Inadequate response
PASDAS: Psoriatic Arthritis Disease Activity Score
PASI: Psoriasis Area and Severity Index
PASI75: ≥ 75% improvement in Psoriasis Area and Severity Index
PBO: Placebo
PsA: Psoriatic arthritis
Q8W: Every 8 weeks
SAA: Serum amyloid A
SD: Standard deviation
SF-36 PCS: 36-item Short-Form Health Survey physical component summary
sICAM: Soluble intercellular adhesion molecule-1
SJC: Swollen joint count
sVCAM: Soluble vascular cell adhesion molecule
Th17: T helper 17
TJC: Tender joint count
TNF: Tumor necrosis factor
TNFi: Tumor necrosis factor inhibitor
TNFi-IR: Tumor necrosis factor inhibitor-inadequate response
