# Correction: Effect of guselkumab on serum biomarkers in patients with active psoriatic arthritis and inadequate response to tumor necrosis factor inhibitors: results from the COSMOS phase 3b study

**DOI:** 10.1186/s13075-023-03158-9

**Published:** 2023-09-15

**Authors:** Georg Schett, Warner Chen, Sheng Gao, Soumya D. Chakravarty, May Shawi, Frederic Lavie, Miriam Zimmermann, Mohamed Sharaf, Laura C. Coates, Stefan Siebert

**Affiliations:** 1 Department of Medicine 3 – Rheumatology and Immunology, Universitatsklinikum Erlangen, Erlangen, Germany; 2 Immunology, Janssen Research & Development, LLC, Spring House, PA USA; 3 Immunology, Janssen Scientific Affairs, LLC, Horsham, PA USA; 4 Drexel University College of Medicine, Philadelphia, PA USA; 5 Immunology, Global Medical Affairs, Janssen Pharmaceutical Companies of Johnson & Johnson, Horsham, PA USA; 6 Immunology, Global Medical Affairs, Janssen Pharmaceutical Companies of Johnson & Johnson, Issy Les Moulineaux, France; 7 Immunology, Janssen Medical Affairs, LLC, Zug, Switzerland; 8 Johnson & Johnson MEA, Dubai, United Arab Emirates; 9 Nuffield Department of Orthopaedics, Rheumatology and Musculoskeletal Sciences, University of Oxford, Oxford, UK; 10 School of Infection and Immunity, University of Glasgow, Glasgow, UK

**Correction: Arthritis Res Ther 25, 150 (2023)**

**https://doi.org/10.1186/s13075-023-03125-4**

Following publication of the original article [1], the authors reported that the corrections were not carried out during the revisions.

The corrections are provided below and the changes have been set in **bold typeface.**

***Author details:***

1 **Department of Medicine 3 – Rheumatology and Immunology**, Universitatsklinikum Erlangen, Erlangen, Germany.

***Abstract***

***Methods***

Adults with active PsA (≥ 3 swollen joints, ≥ 3 tender joints) and IR to one or two TNFi (TNFi-IR) were randomized 2:1 to guselkumab at Weeks 0, 4, then every 8 weeks (Q8W) or placebo➔guselkumab Q8W at Week 24 with possible early escape at Week 16. Levels of serum cytokines, including interferon **γ** (IFN**γ**), IL-10, and tumor necrosis factor α (TNFα); T helper 17 (Th17) effector cytokines IL-17A, IL-17F, and IL-22; and acute phase proteins C-reactive protein (CRP), IL-6, and serum amyloid A (SAA), were assessed and compared with **demographically **matched healthy controls; guselkumab pharmacodynamics through Week 24 were also assessed.

***Results***

Baseline serum levels of IL-6, IL-10, IL-17A, IL-17F, IL-22, TNFα, and IFN**γ** were significantly higher in COSMOS TNFi-IR participants than in healthy controls…

…Guselkumab-treated participants achieving ACR20 response at Week 24 exhibited higher baseline IL-22 and IFN**γ **levels versus nonresponders…

***Keywords***

Serum biomarkers, **IL-23/IL-17** pathway, Guselkumab, Psoriatic arthritis

***Background***

**…** Treatment recommendations from the Group for Research and Assessment of Psoriasis and Psoriatic Arthritis have highlighted interleukin (IL)-23 inhibitors (IL-23i), along with TNFi, IL-17i, and IL-12/IL-23i, as appropriate for use in both biologic-naive and biologicexperienced patients with **peripheral joint symptoms**…

…The objectives of this analysis were to evaluate baseline serum levels of proinflammatory biomarkers in participants with TNFi-IR PsA in the COSMOS trial in comparison **with** healthy controls and **the** relationship **of these biomarker levels with**…

***Clinical assessments***

…ACR20 response was used to assess efficacy in **the** joints at Week 24. Skin responses were assessed among participants with ≥ 3% BSA and Investigator’s Global Assessment (IGA) **score** ≥ 2 [22] at…To assess consistency of response in the biomarker cohort versus **the** overall COSMOS population, additional efficacy outcomes **at Week 24** determined in the biomarker cohort **were** ≥ 50% improvement by ACR criteria (ACR50) response, ≥ 75% improvement in PASI **(PASI75)**, change…

***Biomarker sample collection***

In COSMOS, blood samples for biomarker **analyses** were collected from all participants at Weeks 0, 4, 16, 24, and 48 into standard serum separation tubes…

Serum samples from 24 healthy control **volunteers** (defined as those with no signs of active inflammation,…

***Biomarker analyses***

Serum samples for biomarker **analyses** were analyzed using qualified antibody-based assays…. Acute phase proteins and markers of inflammation, CRP, serum amyloid A (SAA), IL-6, IL-10, interferon **γ** (IFN**γ**), tumor necrosis factor α (TNFα), soluble…

***Statistical analyses***

This analysis included **participants with available baseline values and follow-up** biomarker and clinical data over time. All analyses were post hoc; thus, reported *p* values are nominal….

***Analysis of baseline serum biomarker levels and correlation with disease activity***

…Differences in baseline **serum cytokine** levels between participants with PsA and healthy controls were assessed **using** log2-transformed data **with** a general linear model…

…Correlations between baseline **serum biomarker** levels and baseline disease activity (i.e., DAPSA **scores**, PASDAS, and PASI scores) were assessed using Spearman linear regression, with a Spearman correlation (*rho*) > 0.25 and *p* < 0.05 considered significant…

***Analysis of association between biomarker levels and clinical response***

…Differences in baseline biomarker levels by clinical response at Week 24 (**i.e., response versus nonresponse for ACR20/50, IGA 0/1, and PASI75**)….

***Baseline serum levels and correlation with baseline disease activity***

…Baseline CRP, SAA, and IL-6 levels were positively associated with baseline joint disease severity as measured by the DAS28-CRP (*p* ≤ 0.0001 for each **biomarker**)….

…IL-17A levels showed a trend towards **a** correlation with PASDAS. No statistically significant correlations were observed between baseline levels of other biomarkers evaluated **and** measures of baseline disease activity assessed (Table 1)…

***Effect of treatment on biomarker levels***

In participants **randomized to** guselkumab, reductions from baseline in the **levels of…** Th17 effector cytokines IL-17A, IL-17F,…..

…biomarkers at Week 48 were similar to those observed in participants receiving guselkumab Q8W from **baseline** (Fig. 2)….

***Discussion***

**…**These findings were generally consistent with results from an exploratory biomarker analysis [16] in **patients with active PsA** from the DISCOVER-1 and -2 trials [13, 14]….

…The present study extends these findings by demonstrating that the pharmacodynamic effect **of guselkumab** is sustained through Week 48 in the COSMOS TNFi-IR population, with levels of IL-17F, IL-22, CRP, and SAA approximating the levels seen in healthy controls at Week 48….

…The current limited data on predictors of response across biomarker studies make it difficult to incorporate precision medicine in **the management of** PsA at this stage….

…While analysis of serum levels allows for collection of serial samples in the clinic, evaluation of tissue would further our knowledge **of** disease pathogenesis and could potentially help further elucidate the mechanism(s) of action of guselkumab in joints, as preclinical evidence suggests that IL-17 is a key mediator of PsA joint pathogenesis [40]….

***Figure captions***

Fig. 1 Baseline levels of serum cytokines (IL-10, TNFα, and IFN**γ**), Th17 effector cytokines (IL-17A, IL-17F, and IL-22)…

Fig. 2 **Serum** levels of IL-17A, IL-17F, IL-22, CRP, SAA, and IL-6 in participants with TNFi-IR PsA from COSMOS compared with healthy controls over time….

Fig. 3 Baseline serum cytokine levels and clinical response at Week 24 in participants with TNFi-IR PsA from COSMOS by ACR20 response or IGA 0/1 response and change from baseline in serum IL-6 level by ACR20 or IGA 0/1 response over time. **(A) **Baseline serum levels of IL-22 and IFNγ levels by ACR20 response at Week 24; (**B**) Baseline serum levels of SAA, IFNγ, IL-17A, and IL-6 by IGA 0/1 response at Week 24; (**C**) Change from baseline in IL-6 levels by ACR20 and IGA 0/1 responses through Week 24. *p < 0.05 between responders and nonresponders. ACR20, ≥ 20% improvement **in** American College of Rheumatology **response** criteria;…

***References***

3. Singh JA, Guyatt G, Ogdie A, Gladman DD, Deal C, Deodhar A, et al. Special article: 2018 **American College of Rheumatology/National Psoriasis Foundation** guideline for the treatment of psoriatic arthritis. Arthritis Rheumatol. 2019;71(1):5–32.

4. Coates LC, Soriano ER, Corp N, Bertheussen H, CallisDuffin K, Campanholo CB, et al. **Group for Research and Assessment of Psoriasis and Psoriatic Arthritis** (GRAPPA): updated treatment recommendations for psoriatic arthritis 2021. Nat Rev Rheumatol. 2022;18(8):465–79.

6. Glintborg B, Ostergaard M, Krogh NS, Andersen MD, Tarp U, Loft AG, et al. Clinical response, drug survival, and predictors thereof among 548 patients with psoriatic arthritis who switched tumor necrosis factor **α **inhibitor therapy: results from the Danish Nationwide DANBIO Registry. Arthritis Rheum. 2013;65(5):1213–23.

8. Saad AA, Ashcroft DM, Watson KD, Hyrich KL, Noyce PR, Symmons DP, et al. Persistence with anti-tumour necrosis factor therapies in patients with psoriatic arthritis: observational study from the **British Society of Rheumatology Biologics Register**. Arthritis Res Ther. 2009;11(2):R52.

24 **Ware JE Jr**, Sherbourne CD. The MOS 36-item short-form health survey (SF-36). I. Conceptual framework and item selection. Med Care. 1992;30(6):473–83.

31. Alenius GM, Eriksson C, **Rantapaa Dahlqvist S**. Interleukin-6 and soluble interleukin-2 receptor alpha-markers of inflammation in patients with psoriatic arthritis? Clin Exp Rheumatol. 2009;27(1):120–3.

The original article [1] has been updated.
