# Supplementary material for: Effect of guselkumab on serum biomarkers in patients with active psoriatic arthritis and inadequate response to tumor necrosis factor inhibitors: results from the COSMOS phase 3b study
Source: Arthritis Res Ther. 2023 Aug 16;25:150. doi: 10.1186/s13075-023-03125-4 (PMC10428525; doi:10.1186/s13075-023-03125-4)
Supplement: Supplementary file 1 — Additional file 1: Supplementary Table 1. Baseline demographics and disease characteristics. Supplementary Table 2. Primary and secondary efficacy endpoint results in the COSMOS biomarker population. [file 13075_2023_3125_MOESM1_ESM.docx]

**SUPPLEMENTAL MATERIALS**

**Supplementary Table 1.** Baseline demographics and disease characteristics

|  |  | **COSMOS biomarker population** | | |
| --- | --- | --- | --- | --- |
| **Parameter, mean (SD)^a^** | **All COSMOS participants (N = 285)** | **All participants**  **(N = 150)** | **Guselkumab (N = 100)** | **Placebo  (N = 50)** |
| ***Demographic*** | | | | |
| Age, years | 49.1 (12.2) | 48.4 (12.2) | 48.7 (12.3) | 47.7 (12.2) |
| Male, % | 48.4 | 48.0 | 45.0 | 54.0 |
| BMI, kg/m^2^ | 29.6 (6.4) | 29.3 (6.4) | 28.9 (5.6) | 30.2 (7.7) |
| ***Baseline disease characteristics*** | | | | |
| SJC (0−66) | 9.8 (6.4) | 9.9 (6.3) | 10.3 (6.3) | 9.0 (6.3) |
| TJC (0−68) | 20.1 (12.5) | 19.7 (12.8) | 20.8 (13.6) | 17.7 (10.9) |
| Psoriatic BSA | 16.4 (20.4) | 16.4 (18.7) | 17.7 (18.9) | 13.6 (18.2) |
| IGA score (0−4) | 2.2 (1.0) | 2.2 (0.9) | 2.2 (0.9) | 2.1 (0.9) |
| PASI score (0−72) | 10.8 (11.2) | 10.9 (10.2) | 11.5 (10.2) | 9.7 (10.1) |
| CRP, mg/dL | 1.2 (2.2) | 1.2 (1.8) | 1.3 (2.1) | 1.1 (1.2) |
| PsA disease duration, years | 8.4 (7.6) | 8.7 (7.7) | 9.1 (8.4) | 8.1 (6.2) |
| PsO diagnosis to PsA diagnosis, years | 10.3 (11.2) | 10.5 (11.6) | 11.4 (12.2) | 8.6 (9.9) |
| ***Prior and baseline medication use (% of participants)*** | | | | |
| 1 prior TNFi | 88.4 | 88.7 | 89.0 | 88.0 |
| 2 prior TNFi | 11.6 | 11.3 | 11.0 | 12.0 |
| Methotrexate | 54.4 | 56.7 | 56.0 | 58.0 |
| csDMARDs | 63.2 | 66.7 | 65.0 | 70.0 |
| NSAIDS | 54.4 | 54.7 | 54.0 | 56.0 |
| Oral corticosteroids | 18.9 | 22.0 | 19.0 | 28.0 |

^a^Unless otherwise specified.

BMI, body mass index; BSA, body surface area; CRP, C-reactive protein; csDMARDs, conventional synthetic disease-modifying antirheumatic drugs; IGA, Investigator’s Global Assessment; NSAIDs, nonsteroidal anti-inflammatory drugs; PASI, Psoriasis Area and Severity Index; PsA, psoriatic arthritis; PsO, psoriasis; SD, standard deviation; SJC, swollen joint count; TJC, tender joint count; TNFi, tumor necrosis factor inhibitor.

**Supplementary Table 2.** Primary and secondary efficacy endpoint results in the COSMOS biomarker population

| **Endpoint^a^** | **Guselkumab  (N = 100)** | **Placebo  (N = 50)** | ***p* value** |
| --- | --- | --- | --- |
| ***Primary*** |  |  |  |
| ACR20, % | 44.0 | 20.0 | 0.0068 |
| ***Secondary*** |  |  |  |
| HAQ-DI, mean (SD) change from baseline | −0.22 (0.52) | −0.03 (0.38) | 0.0185 |
| ACR50, % | 19.0 | 6.0 | 0.0605 |
| SF-36 PCS, mean (SD) change from baseline | 4.11 (5.9) | 1.03 (5.17) | 0.0021 |
| IGA 0/1 response, %^b^ | 50.7^c^ | 7.4^d^ | <0.001 |
| PASI75, % | 57.0 | 6.0 | <0.0001 |

^a^Assessed at Week 24; ^b^Among patients with ≥3% body surface area with PsO and IGA score ≥2 at baseline; ^c^N=71; ^d^N=27.

ACR20/50, ≥ 20%/50% improvement by American College of Rheumatology criteria;
HAQ-DI, Health Assessment Questionnaire–Disability Index; IGA, Investigator’s Global Assessment; IGA 0/1 response, Investigator’s Global Assessment score of 0 or 1 with at least a 2-point improvement from baseline; PASI75, ≥ 75% improvement in Psoriasis Area and Severity Index; SD, standard deviation; SF-36 PCS, 36-item Short-Form Health Survey physical component summary.
